# Impact of Encapsulated Thyme, Clove, and Sage Essential Oils on the Antioxidant, Physicochemical, DNA Binding, and Cleaving Properties of Chewing Gum

**DOI:** 10.1021/acsomega.5c02317

**Published:** 2025-07-09

**Authors:** Busra Barman, Yasemin Budama-Kilinc, Gozde Kutlu, Atefeh Karimidastjerd, Arezou Habibzadeh Khiabani, Bahar Gok, Bulent Basyigit, Ibrahim Palabiyik, Nevzat Konar, Fatih Tornuk, Omer Said Toker

**Affiliations:** 1 Chemical and Metallurgical Engineering Faculty, Food Engineering Department, 52999 Yildiz Technical University, Istanbul 34210, Turkey; 2 Chemical and Metallurgical Engineering Faculty, Bioengineering Department, Yildiz Technical University, Istanbul 34210, Turkey; 3 Health Biotechnology Joint Research and Application Center of Excellence, Istanbul 34220, Turkey; 4 Faculty of Fine Arts, Design and Architecture, Department of Gastronomy and Culinary Arts, 566936 Ankara Medipol University, Ankara 06570, Turkey; 5 Engineering Faculty, Food Engineering Department, Harran University, Şanlıurfa 63510, Turkey; 6 Agriculture Faculty, Food Engineering Department, Tekirdag Namik Kemal University, Tekirdag 59300, Turkey; 7 Agriculture Faculty, Dairy Technology Department, 37504 Ankara University, Ankara 06110, Turkey; 8 Faculty of Health Sciences, Department of Nutrition and Dietetics, Sivas Cumhuriyet University, Sivas 58140, Turkey

## Abstract

The use of essential oils (EOs) in various food matrices has gained interest due to their olfactory properties, as well as their antimicrobial and antioxidant activities, making them valuable in the development of functional foods. In this study, thyme, clove, and sage EOs were nanoencapsulated using the ionic gelation method. Their encapsulation efficiency, DNA binding and cleavage properties, particle size, and zeta potential were evaluated. These encapsulated EOs were then incorporated into sugar-free chewing gum formulations, and their antioxidant, anticarcinogenic, sensory, textural, and color properties were analyzed. The results showed the highest encapsulation efficiency for thyme EO (61.51% ± 1.52), followed by clove (56.10% ± 1.87) and sage (52.59% ± 0.56). The average particle sizes (Z-average) of encapsulated thyme, clove, and sage EOs were 330.1 ± 10.89, 452.6 ± 5.52, and 553.7 ± 0.01 d nm, respectively. All encapsulated EOs possessed negative zeta potential values ranging from −26.40 to −32.30 mV and polydispersity index (PDI) values between 0.399 and 0.433. The antioxidant activity of chewing gums increased with the addition of nanocapsules, and the percentage of DPPH inhibited the lowest for control with 6.73% ± 0.32 and 40.41% ± 0.07 for thyme in sugar-free gums. The concentrations of 0.05, 0.1, and 0.2% (w/w) were applied to evaluate the hydrolytic and oxidative cleavage activities. The DNA cleavage experiment revealed that chewing gum samples containing sage EO exhibited hydrolytic and oxidative cleavage activities. In contrast, chewing gum samples containing clove and thyme EOs exhibited only hydrolytic cleavage activity. The addition of 0.2% (w/w) of encapsulated thyme, clove, and sage EOs did not negatively affect the sensory and textural properties of the gum samples. Ultimately, the findings demonstrate that the incorporation of encapsulated EOs successfully enhanced the antioxidant properties of sugar-free gum without compromising its sensory quality.

## Graphical abstract

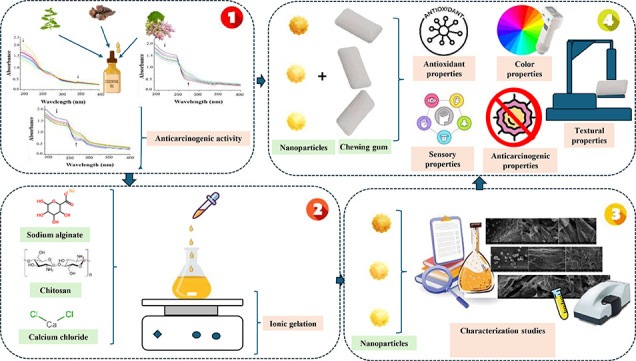

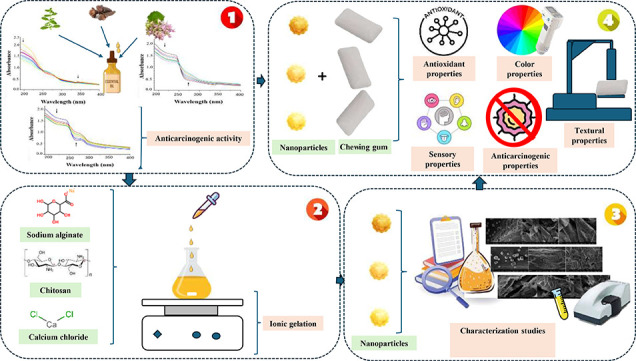

## 1 Introduction

Chewing gum has long been used to freshen breath and cleanse the mouth, with its use spanning centuries.^1^ Today, it is widely consumed for enjoyment across all age groups, but it has also gained attention for therapeutic applications, such as promoting oral hygiene and serving as an alternative for smoking cessation. Consequently, the development of health-promoting chewing gums has become a significant focus within the food industry.^2^ The global chewing gum market is substantial, with an estimated annual production of 560,000 tons and a value of approximately 5 billion US dollars, amounting to the sale of around 374 billion sticks each year.^3^ Chewing gum consists of two key phases: a water-soluble phase that contains flavors, colorants, sweeteners, and preservatives, and a nonsoluble phase, or gum base, made of hydrophilic and hydrophobic polymers that contribute to its texture and functionality.^4^

In recent years, chewing gum has been attracted great attention as a carrying material for bioactive compounds.^5,6^ However, maintaining and protection of the bioactivity under processing conditions is an important issue during production of functional chewing gums. In order to protect the bioactive components from excess environmental conditions, encapsulation is an efficient with many other advantages including masking undesirable tastes and odors of the compounds, improving their physical and/or chemical profitability and preventing losses due to evaporation.^7^ In a variety of encapsulation techniques; the properties of the active substance used, the size of the capsules desired to be obtained, the release rate and amount, economic conditions and production amount are affecting factors. Spray drying, spray cooling, ionic gelation, coacervation, lyophilization and emulsification are examples of the most commonly used encapsulation methods (Table 1).^8^ Encapsulated essential oils are commonly used in food systems to enhance stability, mask undesirable flavors, and enable controlled release. During chewing, their release is influenced by the encapsulation matrix, processing conditions, and the oil’s physicochemical properties. Mechanical mastication and saliva-triggered hydration initiate the rupture or swelling of the encapsulating wall, releasing the oil into the mouth.^9,10^ An initial burst release typically occurs due to surface-located oil and porous capsule structure,^10^ followed by a diffusion-controlled phase governed by Fickian kinetics, as the matrix swells.^11^ Hydrophilic wall materials such as gum Arabic, maltodextrin, or cyclodextrins enhance this diffusion in the moist oral environment. For example, cinnamon oil microcapsules show moisture-dependent solubility, affecting their release profile.^12^

**Table 1:** Various Encapsulation Methods With Each Method’s Efficiency, Advantages and Limitations

Method	Encapsulation Efficiency	Advantages	Limitations	Reference
Spray Drying	40–90%	Economical, scalable	Thermal degradation risk	^13^
Coacervation	>90%	High EE, gentle process	pH-sensitive, complex	^14^
Nano/Microemulsion	>95%	High bioavailability	Surfactant toxicity	^15,16^
Liposomes	30–75%	Biocompatible	Prone to leakage	^17^
Cyclodextrin Complex	40–85%	Improves solubility, masks odor	High cost	^18^
Freeze-Drying	60–90%	Ideal for heat-sensitive EOs	Expensive and time-consuming	^19^

In complex systems like chewing gum, mastication increases surface exposure and erodes capsule walls, enhancing controlled oil release.^20^ This prevents an overwhelming sensory burst and reduces risks like irritation. Ensuring consumer safety involves tuning release profiles to avoid toxic concentrations, particularly for potent oils.^9^ Wall materials such as whey protein and alginate, recognized as safe, support biocompatibility and reduce cytotoxicity.^11^ Moreover, in vitro digestion models simulate oral and gastric conditions to evaluate release kinetics, enabling safe formulation designs and preventing premature release or degradation of the oils.^9,20^ Essential oils (EOs) have been employed for centuries to address various health conditions, and their popularity has risen due to demonstrated safety and efficacy. Numerous *in vitro, in vivo,* and clinical studies have highlighted the antioxidant properties of EOs derived from medicinal plants, especially in promoting oral health.^21^ Among these, thymol, a phenolic monoterpene and isomer of carvacrol, is a major bioactive component of the EO from *Thymus vulgaris* L. (thyme), a Mediterranean medicinal plant renowned for its therapeutic applications. Thymol has attracted significant attention in the pharmaceutical, food, and cosmetic industries due to its antimicrobial, antioxidant, anti-inflammatory, and antispasmodic activities.^22^ Similarly, clove EO, extracted from the buds of *Syzygium aromaticum* (Myrtaceae family), has been widely utilized not only in the flavoring, fragrance, and cosmetics sectors but also for its medicinal benefits. The oil, primarily composed of eugenol, β-caryophyllene, and eugenyl acetate, exhibits notable analgesic, anti-inflammatory, antioxidant, and hepatoprotective properties.^23^ Another valuable EO is extracted from *Salvia sclarea* L., commonly known as clary sage. This plant, from the Lamiaceae family, is widely cultivated for its EO in temperate and subtropical regions. Historically prized for its aromatic and medicinal properties, *S. sclarea* EO is currently prominent in the perfume and cosmetics industries and possesses antibacterial, anti-inflammatory, antioxidant, and anticarcinogenic activities.^24^ These oils were chosen based on their richness in phenolic terpenes (e.g., thymol, eugenol, and carvacrol), which are responsible for both their strong bioactivity and intense and specific aromas. In addition, these EOs are economic, widely available, and generally accepted by consumers in various food applications.^25^ Thyme contains volatile organic compounds: thymol (41.7%) > γ-terpinene (16.0%) > p-cymene (13.0%) > β-caryophyllene (4.7%) > carvacrol (4.0%) > β-bisabolene (2.7%) > α-terpinene (2.6%).^26^ Eugenol is the major compound, accounting for at least 50% in clove EO and the remaining 10–40% consists of eugenyl acetate, β-caryophyllene, and α-humulene.^27^ Sage EO is mainly characterized with 1,8-cineole, camphor, α-thujone, β-thujone, borneol, viridiflorol, and manool, while α-pinene, 1,8-cineol, (+)-camphor, and piperitone are of the reported compounds for rosemary.^28^ In the development of functional products, chitosan and sodium alginate are widely recognized for their roles in encapsulation and controlled release. Chitosan, derived from chitin, offers antimicrobial, antioxidant, and anticarcinogenic properties, making it useful in food and medical industries.^15^ Sodium alginate, a polysaccharide frequently used in food coatings, forms gels by binding with divalent metal ions (Ca^2^^+^, Mg^2^^+^, Sr^2^^+^), which enhances its application in encapsulation, wound dressings, and various biomedical uses. The synergistic combination of chitosan and alginate improves the stability and functionality of encapsulated compounds.^7,25^

In the light of this information, in this study, thyme, clove, and sage EOs were nanoencapsulated using the ionic gelation method and the encapsulation efficiency (%), DNA binding and cleavage, particle size, and zeta potential of the nanocapsules were evaluated. Additionally, sugar-free chewing gums were fortified with these capsules and antioxidants, anticarcinogen, sensory, textural and color properties were analyzed.

## 2 Materials and Methods

### 2.1 Materials

The essential oils (EOs) of *Syzygium aromaticum, Thymus vulgaris* and *Salvia sclarea* were obtained from Bade Natural (Eyüp, Istanbul, Türkiye) while the gum base from Remik Kimya (Pendik, Istanbul, Türkiye). Additionally, sodium alginate, chitosan, CT-DNA, and ethidium bromide were purchased from Sigma (Sigma-Aldrich, Germany). All the chemicals used in this study were reagent grade.

### 2.2 Determination of the Anticarcinogenic Activity of Essential Oils through DNA Binding Studies

The anticarcinogenic activities of thyme, clove, and sage EOs were assessed by evaluating their DNA binding interactions based on the study by Wang et al. (2019).^29^ UV–vis absorption spectroscopy was employed to measure the DNA binding activities of theseEOs. Specifically, UV–vis absorption titration was conducted to determine the DNA binding capacity of sage, clove, and thyme EOs in a Tris–HCl buffer ([tris­(hydroxymethyl)-aminomethane]) at pH 7.2 and room temperature, in the presence of increasing concentrations of calf thymus DNA (CT-DNA). Essential oils were used at a concentration of 2 mg/mL and CT-DNA was used at a range of 0–100 μM. To account for the absorbance contribution of CT-DNA, equal amounts of CT-DNA were added to both the test solution containing the active compounds and the reference solution. The active compound-DNA mixtures were incubated for 5 min at room temperature and UV–vis spectra were recorded at a wavelength range of 200–500 nm.

### 2.3 Nanoencapsulation by Ionic Gelation Technique

The encapsulation of thyme, clove, and sage EOs was carried out based on the protocol described by Li et al. (2008) with slight modifications.^30^ Solutions of sodium alginate (3 mg/mL), chitosan (0.8 mg/mL), and calcium chloride (CaCl_2_, 3.35 mg/mL) were prepared using distilled water. The pH of the sodium alginate solution was adjusted to 5.1 using 0.1 M HCl, while the chitosan solution’s pH was adjusted to 5.4 using 1% acetic acid. Initially, 10 mL of each EO (thyme, clove, and sage) was emulsified with 10 mL of Tween 80 solution by magnetic stirring at 300 rpm for 2 min. Following this, CaCl_2_ solution was gradually introduced dropwise at a flow rate of 0.2 mL/min using an injection method, with stirring continued for an additional 5 min. In parallel, 10 mL of sodium alginate solution was stirred at 500 rpm, while 2 mL of CaCl_2_ solution was added dropwise at a rate of 0.4 mL/min. Afterward, chitosan solution was added dropwise at a flow rate of 1 mL/min, and the mixture was stirred continuously for 1 h to ensure proper formation of the capsules.

The resulting EO capsules were stored overnight at 4 °C. To further purify the capsules, they were centrifuged at 6500 rpm for 30 min at 4 °C. The sedimented capsules containing the encapsulated EOs were then frozen at – 80 °C for 12 h and lyophilized with a freeze drier at −55 °C and 1hPa for 72 h (Martin Christ, Beta1–8 LSC plus, Osterode am Harz, Germany). And finally, lyophilized nanocapsules were stored at 4 °C for subsequent incorporation into the chewing gum formulations

### 2.4 Characterization of EO nanocapsules

#### 2.4.1 Determination of Encapsulation Efficiency

The encapsulation efficiency of the thyme, clove, and sage EOs were determined by spectrophotometric analysis. A 100 mg sample of lyophilized nanocapsules was weighed and placed into a centrifuge tube. To each tube, 4.9 mL of 1 M HCl solution was added. The mixtures were placed in a boiling ultrasonic water bath for 30 min. After removal from the water bath, the mixtures were cooled to room temperature, and 1 mL of ethanol was added. The samples were then centrifuged at 10,000 rpm for 5 min at room temperature. The absorbance of the supernatants was measured at specific wavelengths corresponding to each EOs: 288 nm for thyme, 270 nm for clove, and 285 nm for sage. The absorbance values were calculated using the following calibration curve equation:

$$y=0.3353x+1.0533(R^{2}=0.99)$$ 1

where *y* is measured absorbance value; *x* is essential oil concentration.

The encapsulation efficiency of the EOs was calculated using the following equation:

$$Encapsulation\;efficiency(\%)=[(A_{T}-A_{0})/A_{T}{]}^{*}100$$ 2

where *A*_*T*_ is theoretical absorbance values of the capsules with known essential oil concentration and *A*_*O*_ is measured absorbance values of the capsules with known essential oil concentration.

#### 2.4.2 Determination of Mean Particle Size and Zeta Potential

The average particle size and zeta potential of the capsules were measured using a Zetasizer (Malvern Instruments Co. Ltd., Worcestershire, UK), as previously described by Celik et al. (2024) for particle size and by Subaşı-Zarbaliyev et al. (2023) for zeta potential.^31,32^ To prepare the samples for measurement, the capsule samples were diluted with distilled water to a concentration of 0.1 mg/mL and sonicated. The zeta potential measurements were conducted at 25 °C, while the mean particle size measurements were performed at 30 °C. In colloidal systems, particles with high absolute zeta potential values (typically > ± 30 mV) exhibit strong electrostatic repulsion, reducing the likelihood of aggregation and enhancing stability. Conversely, low zeta potential values can lead to particle agglomeration, resulting in phase separation or sedimentation.^33^ All measurements were conducted in triplicate to ensure accuracy.

#### 2.4.3 Scanning Electron Microscopy (SEM) Analysis

The procedure was carried out based on the previously reported method by Kutlu et al. (2024).^7^ The micromorphological properties of thyme, clove, and sage EO-loaded capsules, obtained through ionic gelation, were examined using a scanning electron microscope (FE-SEM Quanta FEG 250, FEI, USA). Lyophilized capsules were directly mounted onto a specimen holder, and measurements were conducted at an accelerating voltage of 10.00 kV.

### 2.5 Production of Sugar-Free Chewing Gum

Sugar-free chewing gum was produced using essential oil capsules, prepared through both ionic gelation methods. To produce sugar-free gum containing encapsulated thyme, clove, and sage essential oils, 99.8 g of gum base was first placed into a chewing gum mixer and mixed at 60 °C for 20 min. Subsequently, capsules containing 0.2% essential oil were added to the gum base, and the mixture was further stirred for 10 min. The fluid chewing gum mixture was then flattened between sheets of wax paper using a rolling pin and, after cooling, cut into rectangular shapes of 1 cm × 2.5 cm with a weight of 1.5 g each.

Various analyses were performed to determine the quality characteristics and functional properties of the chewing gums. These analyses included antioxidant activity assays, texture analysis, color measurements, sensory evaluation, and anticarcinogenic activity assessments.

### 2.6 DPPH Free Radical Scavenging Activity and Ferric Reducing/Antioxidant Power Assay (FRAP)

The antioxidant activities of the chewing gums were determined using DPPH radical scavenging and FRAP (Iron­(III) ion reducing antioxidant power) methods. The DPPH radical scavenging activity was evaluated based on the method used by Palabiyik et al. (2018).^34^ First, a 0.06 M DPPH-methanol solution was prepared. To obtain extracts from the chewy gum samples, 2.5 g of each sample was weighed and frozen. The frozen samples were then cut into small pieces, 15 mL of methanol was added, and the samples were centrifuged at 6500 rpm for 40 min at 37 °C. From the resulting extracts, 0.1 mL was transferred into glass tubes. Subsequently, 3.9 mL of DPPH solution was added, and the samples were incubated in the dark for 30 min. Methanol (0.1 mL) was used as the blank. Absorbance values were measured using a UV–vis spectrophotometer (Shimadzu, UV-1800) at 517 nm.

The percentage of radical scavenging activity (RSA%) was calculated using the following formula:

$$RSA[(A_{Control}-A_{Sample})/A_{Control}{]}^{*}100$$ 3

where A_control_ represents the absorbance of the blank, and A_sample_ represents the absorbance of the chewy gum sample at 517 nm. A calibration curve using known Trolox concentrations (0,10, 20, 40, 60, 80, 100 μM) and their corresponding % inhibition values.

For the FRAP analysis, the FRAP solution was prepared by mixing 10 mL of acetate buffer (prepared by dissolving 3.1 g sodium acetate trihydrate in 16 mL acetic acid and diluting to 1000 m with water), 1 mL of TPTZ solution (prepared by dissolving 0.7808 g TPTZ in 250 mL of 40 mM HCl), and 1 mL of FeCl_3_H_2_O solution (prepared by dissolving13.52 mg FeCl_3_ in 25 mL distilled water), following the method described by Kutlu et al. (2024) with minor modifications.^7^ To obtain extracts from the chewy gum samples, 2.5 g of each sample was weighed, frozen, cut into small pieces, and mixed with 15 mL of methanol. The samples were centrifuged at 6500 rpm for 40 min at 37 °C. From the supernatant, 0.1 mL was taken, mixed with 1 mL of methanol and 3 mL of FRAP solution, and incubated at 37 °C for 4 min. Absorbance values were measured at 590 nm using a spectrophotometer. The FRAP results were calculated as Trolox equivalent (mg TE/mL) according to the equation below.

y=4.9819x+0.0263 4

where *y* represents the absorbance value and *x* represents the antioxidant compound concentration.

### 2.7 Determination of Textural Properties

The texture properties of the chewing gums were determined using the method described by Bölük et al. (2021).^35^ Chewing gum samples were cut into cubes of 1 × 1 x 1 cm, and their texture was analyzed using a texture analyzer (TA. HD Plus Stable Micro Systems Texture Analyzer, UK). The tests were performed using a 5 kg load cell and a 5 mm cylindrical probe, applying compression to 0.5 mm in two cycles. Parameters such as hardness, adhesiveness, cohesiveness, springiness, chewiness, and resilience were calculated based on the force-time curve. The texture analysis was performed in five replicates.

### 2.8 Color Measurements

The color properties of the chewing gums were measured using a colorimeter (CR-400 Konica, Minolta, Tokyo, Japan). L* (lightness, 0–100), a* (red (+)/ green (−)), and b* (yellow (+)/ blue (−)) values were recorded from different surface regions of each sample. The measurements were performed in triplicate.^13^ The ΔE formula is used to measure the color difference between control (L_1_*, a_1_*, b_1_*) and treated-samples (L_2_*, a_2_*, b_2_*) colors. It is based on the CIELAB color model, L* (lightness), a* (green to red), and b* (blue to yellow). The formula for calculating ΔE is

$$\Delta E=\sqrt{\left(L_{2}^{*}-L_{1}^{*}\right)+\left(a_{2}^{*}-a_{1}^{*}\right)+\left(b_{2}^{*}-b_{1}^{*}\right)}$$

### 2.9 Sensory Analysis

The sensory properties of sugar-free chewing gums were evaluated by a panel of 10 participants using a 9-point hedonic scale (1: unacceptable; 9: excellent). The panelists evaluated the chewing gums for hardness, adhesiveness, color, chewiness, aroma, odor, elasticity, and overall acceptability.^36^ The sensory evaluation was conducted twice with the same panelists. All participants were familiar with chewing gum and reported no aversions or allergies related to chewing or to thyme, clove, or sage. Sensory analysis was conducted in the sensory laboratory at Yildiz Technical University.

### 2.10 Determination of Anticarcinogenic Activity

The anticarcinogenic properties of the chewing gums were assessed by measuring their DNA- cleavage activities. The DNA-cleaving potential was determined using agarose gel electrophoresis with pBR322 plasmid DNA.^37^ The DNA cleavage activities of the chewing gums were evaluated under oxidative conditions in the presence of H_2_O_2_ and under hydrolytic conditions without H_2_O_2_. The experiments were performed by incubating the reaction mixtures (pBR322 DNA, samples containing sage, clove, and thyme essential oil, and water/H_2_O_2_) in Tris-HCl buffer (10 M, pH 7.2) at 37 °C for 3 h. After incubation, the samples were subjected to electrophoresis in TBE buffer (pH 8.0) for 45 min at 80 V, and the bands were visualized under UV light.^38^

### 2.11 Statistical Analysis

The statistical analysis of the data was performed using SPSS Statistics 24 software. Differences between the samples were determined using ANOVA, and significant differences were further analyzed using the Tukey test. A *p*-value of less than 0.05 was considered statistically significant. All analyses were performed in triplicate.

## 3 Results and Discussion

### 3.1 Anticarcinogenic Effects of EOs

Compound possessing antibacterial and anticarcinogenic activity can interact with DNA, the cell’s genetic material, leading to disruptions in its integrity and ultimately causing cell death. This interference can inhibit the growth of pathogenic microorganisms and cancer cells. UV–vis absorption spectroscopy offers a straightforward and efficient way to assess molecule-DNA interactions.^39^ When a molecule binds to DNA, alterations in absorbance and peak shifts are typically observed, indicating the interaction’s strength.^40^

Figures 1a, 1b, and 1c illustrate the DNA-binding activity of clove, thyme, and sage EOs, respectively. The UV–vis spectrum results for sage essential oil indicate a red shift of 3 at 261 nm with a hyperchromic effect of 64.41%, and a red shift of 2 at 240 nm with a hypochromic effect of 54.23%, suggesting significant interaction with CT-DNA. For clove EO, a red shift of 6 at 288 nm was observed along with a hypochromic effect of 29.09% upon successive addition of CT-DNA. In the case of thyme EO, the interaction with CT-DNA resulted in a red shift of 5 at 272 nm with a hyperchromic effect of 46.46%, and a hypochromic effect of 20.58% at 341 nm. Molecules can bind to DNA through electrostatic interactions, groove binding, or intercalative binding.^41^ Redshifts and hypochromic effects in the spectrum are indicative of small molecules binding to DNA via intercalative binding.^42^ The results obtained from the analysis of EOs suggested that thyme, clove, and sage EOs bind to DNA via an intercalative mechanism. A study by Haro-González et al. (2023) utilized a surface response methodology to optimize the formation of nanoemulsions of clove EO.^43^ The resulting nanocapsules demonstrated higher cytotoxic effects against CaCo-2 cancer cells under *in vitro* conditions compared to free clove EO. The essential oils examined in this study (clove, thyme, and sage) are complex mixtures composed of multiple phytochemical constituents, and the molecular weight and exact concentration of each component are unknown. Therefore, classical binding constant (Kb) calculations are not feasible under these conditions, as such analyses require the isolation and characterization of individual active compounds. In this study, the spectroscopic data primarily reflect qualitative observations of changes in absorbance and slight peak shifts upon DNA interaction. It is important to note that, in the literature, many DNA binding studies involving plant-derived extracts or essential oils similarly focus on spectral changes without calculating binding constants. These studies rely on typical patterns of hypochromism and bathochromic shifts to infer interaction characteristics^44,45^ Furthermore, while we acknowledge that calculating the binding constant (Kb) would have quantitatively strengthened our results, it is well-known in the literature that hypochromic effects combined with bathochromic shifts in UV–vis spectra are characteristic signatures of intercalative interactions between small molecules and DNA^46,47^ Investigation of DNA Binding of Newly Designed Zn (II) Complexes With N–N and O–O Donor Ligands as Potential Antioxidants: Spectroscopic, Electrochemical, and Molecular Docking Studies.^46,48^ In our study, both hypochromism and redshifts were consistently observed when CT-DNA was added, supporting the possibility of an intercalative binding mode.

**Figure 1:**
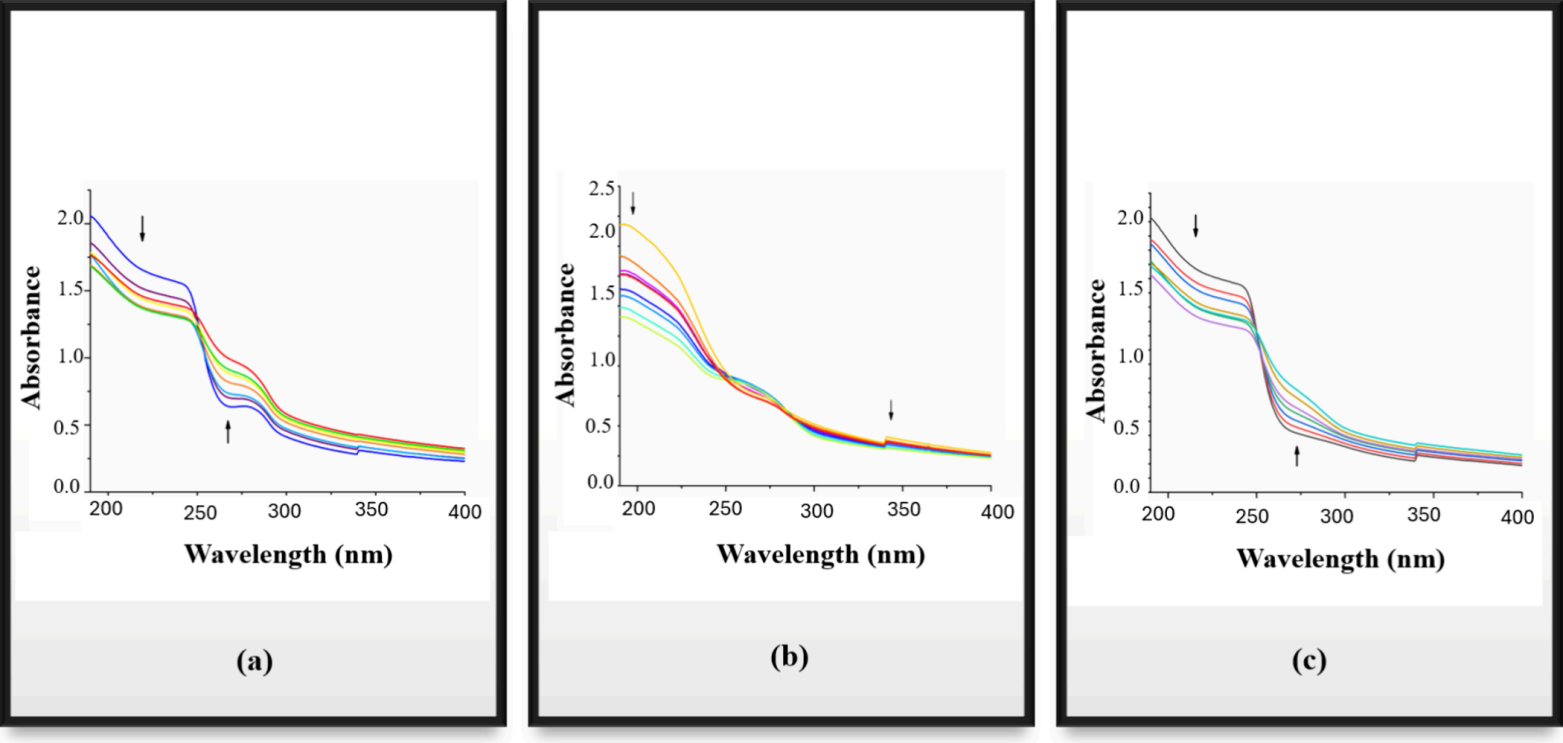
DNA binding spectra of essential oils: (a) Clove oil, (b) Thyme oil, and (c) Sage oil in the absence and presence of increasing concentrations of CT-DNA. Peaks without CT-DNA are shown in blue for clove, yellow for thyme, and gray for sage essential oil. *Arrows (↑↓) indicate changes in absorbance due to increasing DNA concentration.

### 3.2 Characterization of EO Nanocapsules

#### 3.2.1 Determination of Encapsulation Efficiency

Table 2 presents the encapsulation efficiency of EO capsules produced by the ionic gelation method. The results indicated that the highest encapsulation efficiency was achieved for thyme EO encapsulated via the ionic gelation method. The encapsulation efficiency of the nanoencapsulated EOs ranged from 52 to 62%. Notably, no significant difference was observed between the encapsulation efficiencies of EOs when using the same encapsulation method (*P* > 0.05). The ionic gelation method facilitates stabilization during storage by reducing the exposure of the core material to oxygen. In other words, the ionic gelation method provides superior protection for the core material against environmental factors throughout the storage period.^7,22,24^ In a related study, Feyzioglu and Tornuk (2016) reported that the encapsulation efficiency of summer savory EO, encapsulated using the ionic gelation technique, ranged between 35.0% and 40.70%.^48^ They found that the highest encapsulation efficiency was achieved when the initial EO concentration was set at 1.4%, with higher concentrations resulting in reduced efficiency. Similarly, da Rosa et al. (2020) demonstrated that zein nanocapsules loaded with *Origanum vulgare* and *Thymus vulgaris* EOs exhibited encapsulation efficiencies between 80% and 90% using the nanoprecipitation method with a nonionic Pluronic surfactant.^49^ Moreover, Barros et al. (2020) also reported an encapsulation efficiency of 89% for thyme EO encapsulated via spray drying.^50^ In another study, Cui et al. (2015) incorporated clove oil into a liposomal formulation, achieving an entrapment efficiency of 20.41% at a clove oil concentration of 5.0 mg/mL.^51^ Furthermore, Rajaei et al. (2017) explored a coating method by encapsulating clove EO in a chitosan–myristic acid nanogel.^52^ Additionally, Bilenler et al. (2015) encapsulated thyme EO in zein particles using the liquid–liquid dispersion method, with the highest encapsulation efficiency reaching 97.02%.^53^ In addition, Risaliti et al. (2019) reported that liposomes loaded with 100 μL/mL of *Salvia triloba* EO achieved an encapsulation efficiency of 57%.^54^ From a risk assessment perspective, key factors include the concentration of essential oil in the final gum product, the frequency and amount of gum consumed, and the metabolic fate of the oil’s components upon ingestion. It is crucial to compare in-product concentrations with the established acceptable daily intakes (ADI) derived from toxicological studies. If the essential oil present in the gum is significantly lower than the ADI and if the anticipated exposure from habitual use (i.e., chewing frequency and duration) is considered in the risk assessment, then the toxicological risk is minimal. For thyme oil estimated intake can be 220 (μg/person/day).^55^ Nanocapsules added to gum was 0.2% (w/w) that means 2 mg of nanocapsules per 1 g of gum. EO content per mg of nanocapsule (NP), as mentioned EO was used 10 mL which resulted in Thyme EO = 0.001415 mg EO/mg NP, Clove EO = 0.001459 mg EO/mg NP, and Sage EO = 0.001196 mg EO/mg NP.

**Table 2:** Encapsulation Efficiency Values of Various Essential Oil Loaded Capsules^a^

Sample name	Ionic gelation (%)
Thyme	61.51 ± 1.52^a^
Clove	56.10 ± 1.87^a^
Sage	52.59 ± 0.56^a^

a Mean ± standard deviation. The same letters in the column indicate that there is no statistical difference between the values obtained (*P* > 0.05), while the different letters indicate that the difference is statistically significant (*P* < 0.05).

#### 3.2.2 Determination of Mean Particle Size and Zeta Potential of Nanocapsules

Zeta potential (ζ-potential), also known as electrokinetic potential, refers to the electrical potential at the slipping or shear plane of a colloidal particle in an electric field.^56^ It represents the potential difference between the electrophoretically mobile particles and the surrounding dispersing medium, serving as a key indicator of colloidal stability.^57^ Generally, ζ-potential values between −30 and +30 mV indicate stable aqueous suspensions.^7^ Meanwhile, the polydispersity index (PDI) offers insight into particle size distribution uniformity, with lower values closer to zero indicating greater homogeneity.^7,31^

In the present study, the ζ-potential of capsules loaded with thyme, clove, and sage EOs, produced via ionic gelation, was found to be −26.40, −32.30, and −29.00 mV (Table 3), respectively. The PDI values for these capsules ranged from 0.399 to 0.433 (Table 3). While a statistically significant difference in mean particle size (330.10–553.70 nm) was observed among the capsules loaded with various EOs (*P* < 0.05), no significant difference was detected in ζ-potential or PDI values (*P* > 0.05). This finding aligns with the results of Faraji et al. (2020), who encapsulated *Mentha pulegium* and *Ferula gummosa* EOs using nanoliposome technology.^58^ Similar to this study, Faraji et al. (2020) found no statistically significant differences in ζ-potential, mean particle size, or PDI values between the encapsulated oils (*P* > 0.05).^58^ Specifically, the mean particle size of *Mentha pulegium* EO was reported as 345.2 nm, with a PDI of 0.26 and a ζ-potential of −17.2 mV, while *Ferula gummosa* EO had a particle size of 309 nm, a PDI of 0.36, and a ζ-potential of −14.05 mV. In another study by Granata et al. (2021), *Origanum majorana* and thyme EOs were encapsulated using chitosan.^59^ The results indicated that thyme EO had a ζ-potential of +44 mV, while *Origanum majorana* EO had a ζ-potential of +46 ± mV.^59^ In summary, these findings demonstrate consistency in ζ-potential and PDI results across various encapsulation techniques, reaffirming the utility of these measurements in determining the stability and uniformity of EO-loaded particles.

**Table 3:** Average Diameter, PDI, and Zeta Potential Values of Nanocapsules Loaded with Thyme, Clove and Sage Essential Oil Produced by Ionic Gelation Method^a^

Sample name	Z- Ave (d.nm)	PDI	ZP (mV)
Thyme	330.10 ± 10.89^c^	0.399 ± 0.01^a^	–26.40 ± 1.05^a^
Clove	452.60 ± 5.52^b^	0.403 ± 0.04^a^	–32.30 ± 1.27^a^
Sage	553.70 ± 6.01^a^	0.433 ± 0.01^a^	–29.00 ± 2.55^a^

a Mean ± standard deviation. The same letters in the column indicate that there is no statistical difference between the values obtained (*P* > 0.05), while the different letters indicate that the difference is statistically significant (*P* < 0.05).

#### 3.2.3 SEM

Nanocapsules loaded with thyme (Figure a), sage (Figure b), and clove (Figure c) EOs were produced using the ionic gelation method, and their corresponding SEM micrographs are presented in Figure 2. As seen in the images, the morphological structures of the capsules are quite similar, with predominantly spherical shapes, though some cubic particles were also observed. These morphological characteristics are consistent with previous reports in the literature,^48,60^ Furthermore, the particle size distribution of the nanocapsules, as determined by zetasizer measurements, aligns with the morphological findings from the SEM images, reinforcing the consistency between mean particle size and shape. This suggests that the encapsulation process successfully produced uniform particles across the different EOs.

**Figure 2:**
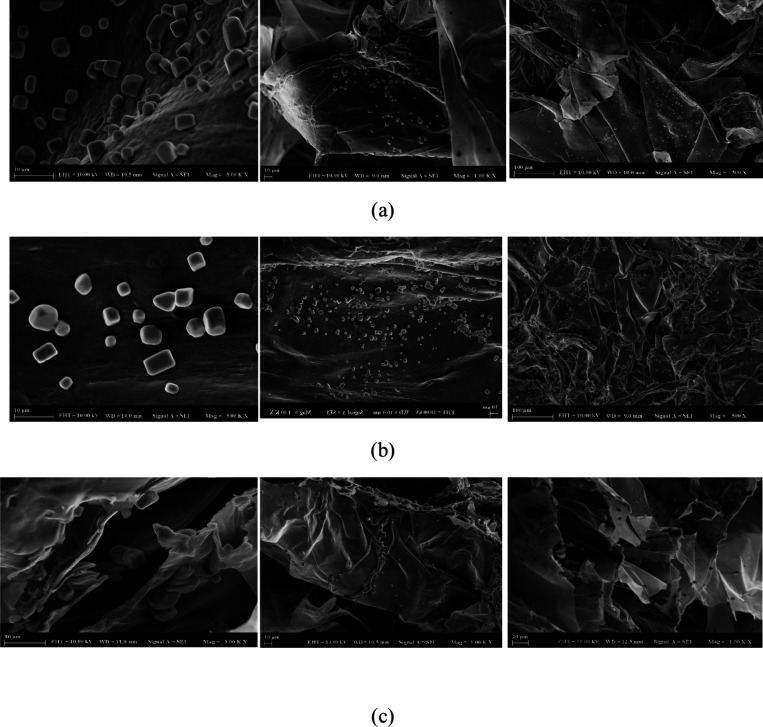
SEM micrographs of nanocapsules loaded with thyme (a), sage (b) and clove (c) essential oils produced by ionic gelation method.

### 3.3 Characterization of Chewing Gum

#### 3.3.1 Antioxidant Activities of Chewing Gum

The antioxidant activities of the chewing gums produced in this study were evaluated using DPPH radical scavenging and Iron­(III) ion reducing antioxidant power (FRAP) assays. The results demonstrated that chewing gums containing EO-loaded capsules exhibited significantly higher radical scavenging activity and iron ion reduction capacity compared to the control samples without EO capsules (CG-C). Specifically, the DPPH radical scavenging activities of sugar-free chewing gums with encapsulated EOs produced via ionic gelation ranged from 12.15% ± 0.01 to 40.41% ± 0.07, while their iron ion reduction capacities varied from 13.38 ± 0.17 to 15.74 ± 0.36 mg/mL (Table 4). Among these samples, the CG-T sample (thyme EO) showed the highest antioxidant activity in both assays. Literature comparisons provide further context for these findings. For instance, Özdoğan (2019) reported DPPH values ranging from 98.83 to 241.89 mg/mL for chewing gums enriched with wheat germ.^61^ Similarly, Palabiyik et al. (2020) found that the antioxidant activities of chewing gums containing microencapsulated pomegranate peel extract were positively correlated with extract concentration, with EC_50_ values ranging from 24.5 to 1540 g sample g^–1^ DPPH.^63^ These studies highlight the influence of encapsulation techniques and bioactive ingredient concentrations on the antioxidant properties of functional chewing gums.

**Table 4:** Antioxidant Activities of Sugar-Free Chewing Gums^a^

Sample	DPPH radical scavenging activity (%)	Fe^2+^-TPTZ concentration(mg TE/mL)
CG-C	6.73 ± 0.32^d^	4.29 ± 0.12^c^
CG-T	40.41 ± 0.07^a^	15.74 ± 0.36^a^
CG-CL	12.15 ± 0.01^c^	13.45 ± 0.04^a^
CG-S	13.97 ± 0.37^b^	13.38 ± 0.17^a^

a **CG-C**: Control sugar-free chewing gum sample without essential oil nanocapsules, **CG-T**: Sugar-free chewing gum sample produced with nanocapsules containing thyme essential oil, **CG-CL**: Sugar-free chewing gum sample produced with nanocapsules containing clove essential oil, **CG-S**: Sugar-free chewing gum sample with flavouring produced with nanocapsules containing sage essential oil. Mean ± standard deviation. The same letters in the column indicate that there is no statistical difference between the values obtained (*P* > 0.05), while the different letters indicate that the difference is statistically significant (*P* < 0.05).

#### 3.3.2 Color Characteristics

The results of color analysis are commonly expressed in terms of L*, a*, and b* values, which represent brightness (L*: 0–100), red-green color spectrum (a*: red (+), green (−)), and yellow-blue color spectrum (b*: yellow (+), blue (−)). As seen in Table 5, the L* values for sugar-free chewy gum produced using the ionic gelation method ranged between 85.97 and 86.50. In terms of the a* values, which reflect the red-green color axis, sugar-free chewy gum produced via ionic gelation showed values ranging from −1.54 to 1.33, indicating a balance between green and red tones. For the b* values, which represent the yellow-blue axis, the sugar-free chewy gum produced by ionic gelation exhibited values between 12.89 and 13.99. The total color difference *(*ΔE*) values provide a measure of overall color variation between the samples. For sugar-free chewy gum produced by ionic gelation, ΔE* values ranged from 0.36 to 2.84. According to Yavuz et al. (2022), color differences with ΔE* values between 1 and 3 are generally not perceptible to the human eye, indicating that the visual color differences observed in these samples are subtle and likely not noticeable by consumers.^63^

**Table 5:** Color Properties of Sugar-Free Chewing Gums^a^

Sample	L*	a*	b*	ΔE*
CG-C	86.27 ± 0.14^ab^	–1.49 ± 0.01^c^	12.89 ± 0.03^b^	-
CG-T	86.50 ± 0.16^a^	1.33 ± 0.02^a^	13.08 ± 0.10^b^	2.84
CG-CL	86.29 ± 0.20^ab^	–1.43 ± 0.02^b^	13.99 ± 0.10^a^	1.10
CG-S	85.97 ± 0.16^b^	–1.54 ± 0.04^c^	13.09 ± 0.20^b^	0.36

a **CG-C**: Control sugar-free chewing gum sample without essential oil nanocapsules, **CG-T**: Sugar-free chewing gum sample produced with nanocapsules containing thyme essential oil, **CG-CL**: Sugar-free chewing gum sample produced with nanocapsules containing clove essential oil, **CG-S**: Sugar-free chewing gum sample with flavouring produced with nanocapsules containing sage essential oil. Mean ± standard deviation. The same letters in the column indicate that there is no statistical difference between the values obtained (*P* > 0.05), while the different letters indicate that the difference is statistically significant (*P* < 0.05).

The addition of thymol or clove EO-loaded capsules to the sugar-free gum formulations led to a slight increase in L* values, suggesting a minor brightening effect; however, this increase was not statistically significant in the clove EO-loaded nanocapsule-containing samples (*P* > 0.05). On the other hand, the addition of sage oil-loaded capsules to sugar-free gum resulted in a slight decrease in L* values compared to the control samples, and this reduction was statistically significant (*P* < 0.05). Regarding the a* values, the incorporation of thymol and clove EO into sugar-free gum formulations caused a statistically significant increase (*P* < 0.05), indicating a shift toward the red spectrum. However, the addition of clove EO-loaded nanoparticles slightly decreased the a* values, although this reduction was not statistically significant (*P* > 0.05). In terms of b* values, a slight increase was observed in the sugar-free gum formulations. This increase was only statistically significant in the samples containing clove EO-loaded particles (*P* < 0.05).

#### 3.3.3 Textural Properties

The texture of sugar-free chewing gum is governed by the complex interactions among its formulation components, particularly bioactive-loaded particles such as EO nanocapsules. These interactions affect not only the mechanical behavior of the gum but also the release kinetics of water-soluble compounds during mastication, ultimately shaping sensory attributes like chewability, elasticity, and mouthfeel—key determinants of consumer satisfaction.^4^

Hardness is a key determinant of consumer acceptance in food products.^63^ In this study, hardness values ranged from 2262.96 ± 338 to 3113.54 ± 147 N (Table 6). The incorporation of EO-loaded nanocapsules tended to decrease hardness, while clove oil nanoencapsulated sample (CG-CL) increased it, although differences were not statistically significant (*p* > 0.05). Higher Hardness in your CG-CL might reflect the added functionality. Mechanistically, softer gums likely result from the plasticizing effect of the nanocapsules, which may disrupt the polymer network, while harder textures may arise from oil–matrix interactions that enhance structural rigidity. While excessively hard gums can reduce palatability, overly soft one’s risk deformation during transport and storage, underscoring the importance of optimizing this parameter.^62^ Springiness, reflecting the gum’s ability to recover its shape after deformation, ranged from 0.55% ± 0.07 to 0.79% ± 0.03. EO-loaded capsules, especially those prepared via ionic gelation, enhanced springiness and adhesion, contributing to a firmer and more elastic chew. This may be attributed to better cross-linking within the gum matrix and the encapsulated oils’ influence on viscoelastic properties. Springiness values are quite varied, with CG-T showing the highest springiness (0.79%), indicating it might be softer or more elastic compared to the others, which might have a more solid or firm texture.^64^ The cohesiveness values of the sugar-free chewy gum samples ranged from 0.66 ± 0.01 to 0.76 ± 0.01 (Table 6). Cohesiveness values show that CG-CL has the highest value, possibly because of stronger interactions or formulation that holds together better under pressure. Cohesiveness, indicating the internal bonding strength of the gum, varied between 0.66 ± 0.01 and 0.76 ± 0.01. The addition of EO-loaded particles significantly increased cohesiveness (*p* < 0.05), likely due to improved matrix uniformity and enhanced intermolecular interactions introduced by the encapsulants. This stronger matrix may help maintain gum integrity over extended chewing periods.^64^ Adhesiveness, defined as the negative force required to detach the gum from a surface (e.g., teeth), ranged from −0.08 ± 0.01 to −0.04 ± 0.01. Although the addition of EO-loaded nanocapsules did not significantly alter adhesiveness (*p* > 0.05), its control remains essential, as excessive stickiness can negatively impact mouthfeel and consumer experience. The textural properties in the sumac gum study by Ostadrahimi et al. (2025) showed firmness decreased with higher doses of freeze-dried sumac extract, though not in a strictly linear manner.^5^ This softening effect is attributed to the extract’s viscous nature and its ability to disperse within the gum matrix and absorb more water during chewing, enhancing gum softening through increased saliva uptake. In our study, CG-CL (gumminess 2344 N, chewiness 1395 N) shows a higher value in terms of gumminess and chewiness, possibly due to the extract’s effects. Chewiness refers to the energy needed to chew a solid food and is determined by the combination of hardness, cohesiveness, and springiness. It describes textures ranging from tender to chewy or tough.^53^ For sugar-free chewy gum, chewiness values ranged from 895.63 ± 134 to 1394.57 ± 96, with an increase observed due to the addition of EO-loaded capsules (*p* < 0.05). No significant differences were found between the types of EOs used, such as thymol and sage (*p* > 0.05). Gumminess, another integrative measure reflecting the energy required to disintegrate a semisolid food, ranged from 1118 ± 155 (CG-T) to 2344 ± 133 (CG-CL), with no significant differences among CG-C, CG-T, and CG-S (*p* > 0.05). Variations in gumminess likely arise from the interplay of encapsulant properties and their effect on structural cohesion.^52^ Resilience, or the gum’s ability to recover its shape after compression, ranged from 0.55 ± 0.03 to 0.76 ± 0.04. Thymol-loaded capsules significantly reduced resilience in CG-T samples, potentially due to matrix softening. Conversely, slight, nonsignificant increases in resilience were observed in CG-CL and CG-S samples, suggesting formulation-dependent mechanical recovery dynamics.

**Table 6:** Texture Properties of Sugar-Free Chewing Gums^a^

Sample	Hardness (N)	Springiness (%)	Adhesiveness	Cohesiveness	Gumminess	Chewiness	Resilience
CG-C	2509 ± 108^a^	0.55 ± 0.07^b^	–0.05 ± 0.01^a^	0.66 ± 0.01^c^	1631 ± 44^b^	896 ± 134^b^	0.66 ± 0.03^ab^
CG-T	2263 ± 338^a^	0.79 ± 0.03^a^	–0.08 ± 0.01^b^	0.72 ± 0.03^b^	1118 ± 155^b^	1290 ± 260^ab^	0.55 ± 0.03^b^
CG-CL	3114 ± 147^a^	0.68 ± 0.02^a^	–0.06 ± 0.00^a^	0.76 ± 0.01^a^	2344 ± 133^a^	1395 ± 96^a^	0.76 ± 0.04^a^
CG-S	2347 ± 31^a^	0.60 ± 0.01^a^	–0.04 ± 0.01^a^	0.72 ± 0.01^c^	1686 ± 35^b^	1143 ± 25^ab^	0.68 ± 0.01^ab^

a **CG-C**: Control sugar-free chewing gum sample without essential oil nanocapsules, **CG-T**: Sugar-free chewing gum sample produced with nanocapsules containing thyme essential oil, **CG-CL**: Sugar-free chewing gum sample produced with nanocapsules containing clove essential oil, **CG-S**: Sugar-free chewing gum sample with flavouring produced with nanocapsules containing sage essential oil. Mean ± standard deviation. The same letters in the column indicate that there is no statistical difference between the values obtained (*P* > 0.05), while the different letters indicate that the difference is statistically significant (*P* < 0.05).

In conclusion, the incorporation of EO-loaded particles significantly influenced key textural parameters, depending on the formulation and type of EO used. These interactions are critical for developing chewing gums with optimal sensory attributes and mechanical stability.

#### 3.3.4 Sensory Evaluation

The sensory evaluation results of sugar-free samples containing encapsulated EOs are presented in Table 7. According to panelists’ assessments, the incorporation of encapsulated EOs into the sugar-free gum formulation did not lead to significant changes in sensory properties such as hardness, adhesiveness, chewiness, aroma, smell, and elasticity, with the only exception being color (*P* > 0.05). This finding suggests that while encapsulated EOs can enhance the functional properties of the chewing gum, their impact on the sensory qualities that directly influence consumer acceptance remains minimal. Such results are important for product formulation as they imply that the inclusion of encapsulated EOs could be used to improve specific characteristics without adversely affecting the overall sensory experience of chewing gum. According to sensory evaluation of panelist, some parameters could be opposite to texture results such as hardness, adhesiveness, chewiness and resilience. The reason is that sensory analysis was carried out in body temperature while texture analysis was done in room conditions.^26^ Based on Principal Component Analysis (PCA) results (Figure 3), we can conclude that general acceptability and adhesiveness align closely with CG-S and CG-CL, indicating that these samples were better received. Color is strongly aligned with CG-C, consistent with your earlier finding that CG-C had the highest color score. Aroma, odor, and elasticity are more central, suggesting they contribute more evenly across all samples. Thyme (CG-T) appears to be influenced by Adhesiveness and Chewiness but shows less color alignment.

**Figure 3:**
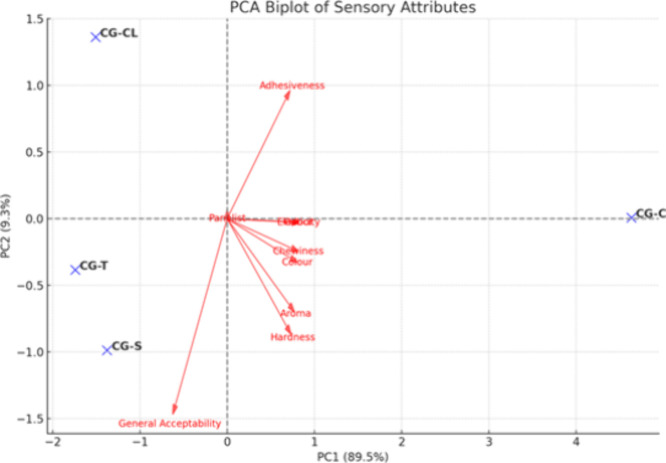
Principal Component Analysis (PCA) for correlation of textural and sensory parameters.

**Table 7:** Sensory Properties of Sugar-Free Chewing Gums^a^

Sensory properties	CG-C	CG-T	CG-CL	CG-S
Hardness	5.63 ± 2.06^a^	5.75 ± 2.87^a^	6.25 ± 0.2.76^a^	5.75 ± 3.01^a^
Adhesiveness	7.13 ± 2.09^a^	7.25 ± 1.39^a^	8.00 ± 0.76^a^	7.00 ± 2.32^a^
Color	8.00 ± 0.71^a^	4.38 ± 1.30^b^	5.63 ± 1.85^a^	5.63 ± 2.07^b^
Chewiness	7.13 ± 1.05^a^	4.88 ± 1.46^a^	5.88 ± 1.96^a^	5.38 ± 2.13^a^
Aroma	6.13 ± 1.54^a^	6.13 ± 2.10^a^	6.88 ± 1.96^a^	6.50 ± 2.51^a^
Odor	7.13 ± 1.62^a^	4.63 ± 1.77^a^	5.88 ± 1.81^a^	5.25 ± 2.25^a^
Elasticity	6.38 ± 2.06^a^	4.88 ± 1.25^a^	6.00 ± 1.60^a^	5.50 ± 2.51^a^
General acceptability	5.25 ± 0.50^a^	7.00 ± 1.15^a^	7.00 ± 1.41^a^	7.25 ± 1.71^a^

a **CG-C**: Control sugar-free chewing gum sample without essential oil nanocapsules, **CG-T**: Sugar-free chewing gum sample produced with nanocapsules containing thyme essential oil, **CG-CL**: Sugar-free chewing gum sample produced with nanocapsules containing clove essential oil, **CG-S**: Sugar-free chewing gum sample with flavouring produced with nanocapsules containing sage essential oil. Mean ± standard deviation: The same letters in the line indicate that there is no statistical difference between the values obtained (*P* > 0.05).

#### 3.3.5 Anticarcinogenic Properties of Chewing Gum

The anticarcinogenic potential of chewing gum samples infused with EOs (sage, clove, and thyme) loaded capsules was evaluated by analyzing nuclease activity via both hydrolytic and oxidative mechanisms. Hydrolytic cleavage breaks the phosphodiester bonds in DNA, while oxidative cleavage affects the deoxyribose sugar or nucleobases.^29^ DNA cleavage activity was assessed by monitoring the gradual degradation of supercoiled DNA into nicked circular and linear forms.^32^ During electrophoresis, the supercoiled form of the pBR322 plasmid DNA migrated the fastest. If a single DNA strand is cleaved, the supercoiled form relaxes into a slower-moving nicked circular form, while further cleavage produces a linear form, which migrates at a speed between the supercoiled and nicked circular forms. In this study, the DNA cleavage experiment aimed to qualitatively evaluate DNA cleavage mechanisms (hydrolytic and oxidative) by observing the conversion of supercoiled DNA into nicked circular and linear forms. Since essential oils were tested within a complex chewing gum matrix and the primary objective was to determine the presence of cleavage activity under different conditions, densitometric analysis of band intensities was not performed.^65,66^ The results revealed that chewing gum samples containing sage EO exhibited both hydrolytic and oxidative cleavage activities, suggesting a dual mechanism of action in DNA degradation (Figure 4). In contrast, chewing gum samples containing clove and thyme EOs demonstrated only hydrolytic cleavage activity, with no oxidative cleavage observed. These findings suggest that sage EO may offer broader anticarcinogenic potential due to its ability to induce both types of DNA cleavage, whereas clove and thyme EOs are more limited in their action, showing only hydrolytic effects. This distinction could be relevant for the formulation of functional chewing gum products with antioxidant and anticancer effects. Sage EO standing out as a promising candidate for further research due to its enhanced cleavage capabilities. Although direct enzymatic assays or ROS quantification were not performed in the current study, the observed oxidative cleavage is consistent with prior reports demonstrating the oxidative potential of sage. For example, Sönmez et al. (2015) observed a significant increase in antioxidant enzyme activities (SOD, G6PD, GPx) and a decrease in lipid peroxidation levels (MDA) in rainbow trout (Oncorhynchus mykiss) supplemented with dietary sage.^67^ These findings demonstrate that sage has potent biological effects on oxidative stress and support the scientific plausibility of oxidative DNA scission observed in our study. In the European Union, essential oils (EOs) used as flavorings in food products, including chewing gum, are regulated under Regulation (EC) No 1334/2008, which mandates that such substances must not pose health risks and should not mislead consumers through their use levels. Certain EO constituents, like thujone found in sage and thyme oils, have established maximum limits due to potential toxicity concerns. Additionally, product labeling must clearly indicate all ingredients and highlight any allergens.^68^ In the United States, several natural antimicrobial substances are recognized as Generally Recognized as Safe (GRAS) under 21 CFR 182. This includes components like lactoferrin, lysozyme, hops beta acids, polylysine, citrus extracts, and bacteriophage preparations. While the GRAS inventory does not separately list antimicrobial agents, many plant-derived (e.g., essential oils, herbs, spices), animal-derived (e.g., chitosan, antimicrobial peptides, lactoperoxidase), and microbial-derived (e.g., bacteriocins, nisin, reuterin) substances meet the criteria for GRAS status and can be used in place of synthetic preservatives.^69^

**Figure 4:**
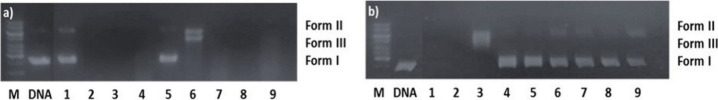
*DNA cleavage activity of fat-containing chewing gums. a) Hydrolytic DNA cleavage, b) Oxidative DNA cleavage (M: Marker, DNA: control, 1: Sage chewing gum 0.05%, 2: Sage chewing gum 0.1%, 3: Sage chewing gum 0.2%, 4: Clove chewing gum 0.05%, 5: Clove chewing gum 0.1%, 6: Clove chewing gum 0.2%, 7: Thyme chewing gum 0.05%, 8: Thyme chewing gum 0.1%, 9: Thyme chewing gum 0.2%).*.

## 4 Conclusions

EOs, prized for their bioactive properties and extensive use across industries, are effectively integrated into chewing gum, which serves as an ideal medium for delivering health benefits due to its prolonged presence in the mouth. This study aimed to develop functional chewing gum formulations enriched with thyme, clove, and sage EOs encapsulated through ionic gelation method, while also characterizing the nanocapsules. The results demonstrated that the antioxidant activity of the chewing gum samples increased when EOs were encapsulated using both ionic gelation method. Moreover, the textural, color, and sensory analysis indicated that the encapsulation of EOs did not adversely affect the quality of the chewy gum samples. The findings indicated that chewing gum samples infused with sage EO loaded capsules demonstrated both hydrolytic and oxidative cleavage activities, implying a dual mechanism for DNA degradation that could aid in anticancer effects, positioning sage EO as a promising candidate. Future research should investigate ways to enhance EO concentrations in gum formulations while maintaining quality, explore different oil sources, and examine the release of active compounds to promote oral health, particularly through antibacterial properties. In light of the observed DNA binding and cleavage activities, further studies, including MTT assays to evaluate both cytotoxic safety and anticancer efficacy, and in vivo experiments to investigate biological significance are planned.

## Data Availability

The data sets obtained during the current study are available throughout the manuscript.
